# *Neisseria chenwenguii* sp. nov. isolated from the rectal contents of a plateau pika (*Ochotona curzoniae*)

**DOI:** 10.1007/s10482-019-01234-2

**Published:** 2019-02-24

**Authors:** Gui Zhang, Jing Yang, Xin-He Lai, Shan Lu, Dong Jin, Ji Pu, Xiangning Bai, Xuelian Luo, Yanwen Xiong, Ying Huang, Cuixia Chen, Jianguo Xu

**Affiliations:** 1 State Key Laboratory of Infectious Disease Prevention and Control, Collaborative Innovation Center for Diagnosis and Treatment of Infectious Diseases, National Institute for Communicable Disease Control and Prevention, Chinese Center for Disease Control and Prevention, Beijing, 102206 People’s Republic of China; 2 Shanghai Institute for Emerging and Re-emerging Infectious Diseases, Shanghai Public Health Clinical Center, Shanghai, 201508 People’s Republic of China; 3 School of Biology and Food Science, Shangqiu Normal University, Shangqiu, 475000 Henan Province People’s Republic of China

**Keywords:** Average nucleotide identity, *Neisseria*, Novel species, Phylogeny

## Abstract

Two Gram-stain negative, catalase positive, coccus shaped bacteria, designated 10023^T^ and 10010, were isolated from the rectal contents of a plateau pika (*Ochotona curzoniae*) in Qinghai–Tibet Plateau, China. Based on 16S rRNA gene sequence analysis, phylogenetic trees showed that these two isolates (10023^T^, 10010) group with members of the genus *Neisseria*. Additionally, these two isolates exhibited high 16S rRNA gene sequence similarity with *Neisseria zalophi* CSL 7565^T^ (96.98%), *Neisseria wadsworthii* WC 05-9715^T^ (96.92%) and *Neisseria canis* ATCC 14687^T^ (96.79%). Further phylogenetic analysis based on the *rplF* gene showed that these two novel strains can be easily discriminated from phylogenetically closely related species. Optimal growth was found to occur on BHI agar with 5% defibrinated sheep blood at 37 °C and growth was also observed on nutrient agar, Columbia blood agar and chocolate agar plates; however, growth was not observed on MacConkey agar after 7 days. The major cellular fatty acids of these strains were identified as C_16:0_ and C_16:1_ω7c/C_16:1_ω6c. The complete genome size of the type strain 10023^T^ is 2,496,444 bp, with DNA G+C content of 54.0 mol %. The average nucleotide identity values were 73.5–79.3% between isolate 10023^T^ and reference *Neisseria* spp. Based on polyphasic analysis, these isolates (10023^T^ and 10010) are considered to represent a novel species in the genus *Neisseria*, for which the name *Neisseria chenwenguii* sp. nov. is proposed. The type strain is 10023^T^ (= DSM 103440^T^ = CGMCC 1.15736^T^).

**Electronic supplementary material:**

The online version of this article (10.1007/s10482-019-01234-2) contains supplementary material, which is available to authorized users.

## Introduction

The genus *Neisseria* is the type genus of the family *Neisseriaceae*. Members of the genus are non-motile Gram-negative cocci, occurring singly, in pairs, or in short chains, with no flagella (Adeolu and Gupta 2013). There are currently 30 species and 3 subspecies in the genus *Neisseria* (http://www.Bacterio.net/Neisseria.html), among which *Neisseria meningitidis* and *Neisseria gonorrhoeae* are well-known human pathogens, causing bacterial meningitis and gonorrhoea, respectively (Virji 2009). Other species in the genus *Neisseria* may be associated with human and animal infections, having been isolated from animals, such as ducks (Murphy et al. 2005), mice (Weyand et al. 2016), sea lions (Volokhov et al. 2018) and clinical specimens or nonviable eggs (Hansen et al. 2017; Wolfgang et al. 2011, 2013; Wroblewski et al. 2017).

The Qinghai–Tibet Plateau, also known as the Third Pole or “the Roof of the World”, is at a high altitude and has a high level of ultraviolet radiation, low oxygen and temperature, and hosts wild animal species such as wild pikas (*Ochotona curzoniae*), snow leopards (*Panthera uncia*), marmots (*Marmota himalayana*), Tibetan antelopes (*Pantholops hodgsonii*) and vultures. Several novel bacterial species have been isolated and characterised from plateau wild animals, including *Helicobacter himalayensis* (Hu et al. 2015), *Escherichia marmotae* (Liu et al. 2015), *Streptococcus halotolerans* (Niu et al. 2016), *Streptococcus himalayensis* (Niu et al. 2017), *Streptococcus pantholopis* (Bai et al. 2016), *Actinomyces liubingyangii* and *Actinomyces vulturis* (Meng et al. 2017a, b).

The wild plateau pika (*O. curzoniae*) is a tiny mammal belonging to the Order *Lagomorpha*, which includes rabbits and hares. The plateau pika is abundant and pika holes can be found all over the grassland, suggesting that the plateau pika functions as a key species on the alpine meadow ecosystem in Qinghai Province, PR China (Hogan 2010). During our research into the microbial diversity in the rectal contents of plateau pika, representatives of a novel species in the genus *Neisseria* were obtained in 2015. Therefore, in this study, we report the isolation and characterisation of this novel *Neisseria* species isolated from plateau pika. Two isolates (10023^T^, 10010) were obtained, from different pika samples, which showed around 97% 16S rRNA gene similarity to several members of the genus *Neisseria*. In this study, the taxonomic position of these two isolates is reported based on the polyphasic approach, including phylogenetic analysis of genes (16S rRNA and *rplF*), average nucleotide identity (ANI), biochemical and chemotaxonomic characterisation. We conclude that these two isolates are distinct from previously described species of *Neisseria* and represent a novel species in this genus, for which the name *Neisseria chenwenguii* sp. nov. is proposed.

## Materials and methods

### Isolation, maintenance and cultivation of *Neisseria* strains

Plateau pikas (*O. curzoniae*) were identified based on genotype (*cox1* and *cox2*, encoding *O. curzoniae* cytochrome c oxidase subunit 1 and 2) and phenotype. They were captured by mouse traps and anaesthetised with ether in Yushu Tibetan Autonomous Prefecture in August 2015, as approved by the ethics committee of the National Institute for Communicable Disease Control and Prevention, China CDC. Colon and rectum samples from hundreds of pikas were collected and aliquoted into 5 mL sterile tubes containing 2 mL 30% (w/v) glycerol buffer. Samples were placed in an icebox before being transported to the local laboratory and stored at − 20 °C, followed by cold chain transportation to deliver the pika samples to our laboratory in Beijing. Isolates (10010 and 10023^T^) were obtained from the rectal contents of two separate plateau pika (4266 meters above sea level, 33°01′46″N, 96°47′08″E, male/adult for 10023^T^, and 4278 m, 33°01′58″N, 96°46′57″E, female/adult for 10010) using BHI agar with 5% defibrinated sheep blood at 37 °C, 5% CO_2_ for two days. Pure cultures were obtained after three successive transfers of single colonies to the same medium. These two novel isolates were preserved at − 80 °C as a suspension in Brain Heart Infusion Broth with 30% (w/v) glycerol.

*Neisseria wadsworthii* DSM 22247^T^ and *Neisseria canis* DSM 18000^T^ were obtained from the Leibniz Institute DSMZ-German Collection of Microorganisms and Cell Cultures and cultured under the same conditions as the reference strains.

### Phylogenetic analysis

Using the Wizard^®^ Genomic DNA Purification Kit, the total DNA were extracted from isolates 10010 and 10023^T^ cultured on BHI agar with 5% defibrinated sheep blood under the condition of 37 °C, 5% CO_2_. Subsequently, the 16S rRNA gene was amplified using the forward primer (5′-AGAGTTTGATCCTGGCTCAG-3′, corresponding to *Escherichia coli* positions 8–27) and the reverse primer (5′-ACGGCTACCTTGTTACGACTT-3′; 1492–1512) (Neilan et al. 1997), with these amplification conditions (a single cycle at 95 °C for 5 min, followed by 30 cycles at 95 °C for 45 s, 54 °C for 45 s and 72 °C for 1.5 min, followed by a final extension at 72 °C for 10 min and at 4 °C hold). A BLAST search based on the EzTaxon database (https://www.ezbiocloud.net/identify) was performed. The almost complete 16S rRNA gene sequences of these two novel strains and the 30 closest taxa identified with validly published names were aligned using the Clustal W program (Thompson et al. 2002). A phylogenetic tree based on the 16S rRNA gene was constructed by different methods (Neighbour-Joining and Maximum-Likelihood) using the MEGA 7.0 software (www.megasoftware.net) (Kumar et al. 2016).

Phylogenetic analysis of the *rplF* gene was also performed as recommended by Bennett et al. (2013, 2014) to better discriminate the novel species from its phylogenetically closely related species. *rplF* gene sequences of these two novel isolates, *Neisseria* spp and other related genera were aligned using the Clustal W program, and a Neighbour-Joining tree was constructed with the MEGA 7.0 software (www.megasoftware.net).

### Average nucleotide identity and genes similarity analysis

The ANI calculation between a given pair of genomes has been used as the gold standard for microorganism classification, and a value of 95% is recommended as the threshold for delineating species (Richter and Rossello-Mora 2009). To reaffirm the relationship of the novel species with other species in the genus *Neisseria*, an ANI calculation was performed using online tools at http://www.ezbiocloud.net/tools.

For the type strain 10023^T^, the complete genome was sequenced using the PacBio sequencing platform (RSII). DNA was initially treated into fragments of appropriate size by g-TUBE. Subsequently, the fragments were damage repaired and ends repaired. Both sides of the DNA fragments were respectively connected with hairpin adapters to creat a dumbbell (set of horse ring) structure, which is known as the SMRTbell. After annealing, the SMRTbell was fixed at the bottom of the ZWM polymerase, for use in the final stage of sequencing. Raw data was assembled by Single Molecule, Real-Time (SMRT) Analysis 2.3.0 (Berlin et al. 2015). The draft genome of isolate 10010 was sequenced using the Illumina PE150 platform and assembled by SOPA denovo. The genomes of these two novel isolates were annotated using the method of best-placed reference protein set and the GeneMarkS+ 4.2 software.

Genes with greater nucleotide sequence diversity than the 16S rRNA gene, including *rplF* (encoding 50S ribosomal protein L6), *argF* (ornithine carbamoyltransferase), *recA* (DNA recombination/repair protein RecA), *rpoB* (DNA-directed RNA polymerase subunit beta), *rpoD* (RNA polymerase sigma factor RpoD), *polI* (DNAdirected DNA polymerase I), *ribII* (ribonuclease II), *aspS* (aspartate-tRNA ligase), *gyrB* (DNA gyrase subunit B) and porin precursor (*por*) exist in all *Neisseria* species (Volokhov et al. 2018). As in previous studies, similarities for these genes between strain 10023^T^ and closely related *Neisseria* type strains were analysed.

### Chemotaxonomic analyses

Test and reference strains were cultured on BHI agar with 5% defibrinated sheep blood plate at 37 °C, 5% CO_2_, and harvested in the late-exponential growth phase. To determine fatty acid content and compositions, five solutions were prepared in advance (solution I: 45 g NaOH in 1:1 distilled water/methanol; solution II: 190 mL concentrated hydrochloric acid/275 mL methanol in 135 mL distilled water; solution III: 1:1 n-hexane/MTBE; solution IV: 10.8 g NaOH in 900 mL distilled water; solution V: saturated sodium chloride). The cellular fatty acids of test and reference strains were saponified, methylated and extracted according to the protocol of the Sherlock Microbial Identification System (MIDI) and analysed by GC (model 6890, Hewlett Packard).

### Morphological, physiological and biochemical analysis

Type strain 10023^T^ was cultivated for 2 days at 37 °C on BHI agar with 5% defibrinated sheep blood for morphological observation using a transmission electron microscope (HT-7700). Gram-staining was performed using the Color Gram 2 kit (bioMérieux, France), according to the manufacturer’s protocol. Motility was examined by inoculation in a semisolid BHI broth. The temperature range for growth was determined using BHI agar with 5% defibrinated sheep blood for a week of incubation at 4–45 °C. Salt tolerance was tested in BHI broth supplemented with 0–8.0% (w/v) NaCl (at 1.0% intervals) after 5 days of incubation at 37 °C. The pH range (pH 3.0–11.0, at 1 pH unit intervals) for growth was determined using BHI broth that was buffered with citrate/phosphate buffer or Tris/hydro-chloride buffer (Wang et al. 2015). Anaerobic growth was tested on BHI agar with 5% defibrinated sheep blood plate using the Anaerobic workstation (ELECTROTEK, Britain), in which the air was substituted with 90% N_2_, 5% H_2_ and 5% CO_2_. *Bacteroides vulgatus* DSM 1447^T^ was used as a positive control. Growth on different media was detected, including nutrient agar, BHI, Columbia blood or chocolate plates and MacConkey agar. Phenotypic characteristics (API NH, API ZYM) were tested strictly based on the manufacturers^’^ instructions with cells cultured on BHI agar with 5% defibrinated sheep blood under the conditions of 37 °C and 5% CO_2_. An oxidase activity test was performed using a commercial dropper oxidase reagent (Becton–Dickinson, USA). Utilisation of Simmons’ citrate was tested using Simmons citrate agar.

## Results and discussion

### Phylogenetic analysis

**Fig. 1:**
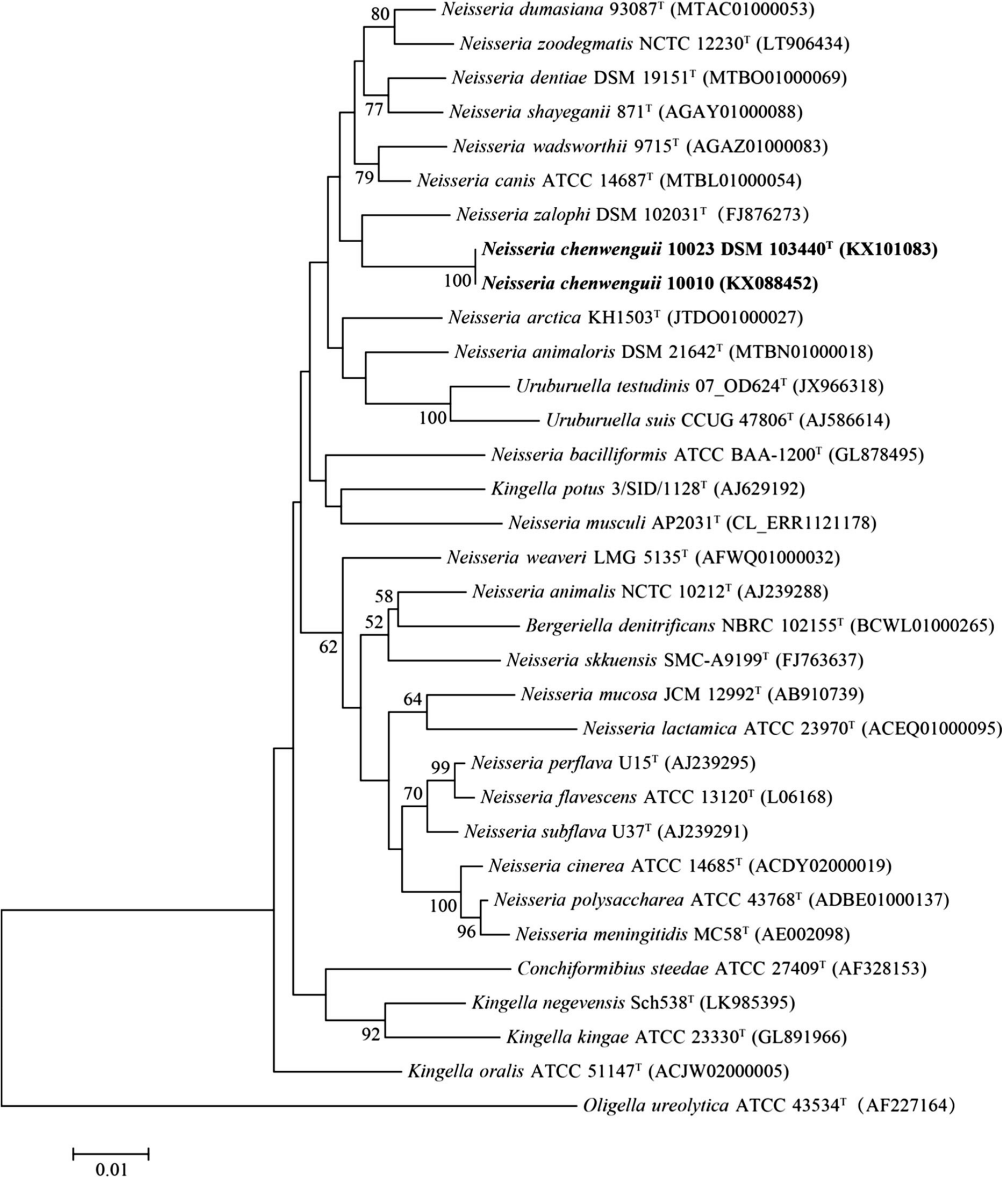
Neighbor-joining tree reconstructed from 16S rRNA gene sequences of *Neisseria chenwenguii* sp. nov. and the 30 closest taxa returned from a BLAST search of the EzTaxon database. The tree was constructed based on the Kimura 2-parameter model conducted in MEGA7.0 rooted with *Oligella ureolytica* DSM 18253^T^. The percentage of replicate trees in which the associated taxa clustered together in the bootstrap test (1000 replicates) are shown next to the branches. The tree is drawn to scale, with branch lengths in the same units as those of the evolutionary distances used to infer the phylogenetic tree. There were a total of 1294 positions in the final dataset. Bar, 0.01 expected changes per site

The sequences of the 16S rRNA genes of isolates 10023^T^ and 10010 were found to be identical. Sequence analysis indicated these two novel strains to be closely related to *Neisseria zalophi* CSL 7565^T^, *N. wadsworthii* WC 05-9715^T^ and *N. canis* ATCC 14687^T^ with similarities of 96.98, 96.92 and 96.79%, respectively. Phylogenetic trees constructed using different methods based on 16S rRNA genes were quite similar (Neighbour-Joining tree for Fig. 1 and Maximum-Likelihood tree for Supplementary Fig. S1). These two novel strains grouped with members of the genus *Neisseria* and reside in an independent clade that harbours 7 *Neisseria* species, including *N. zalophi*, *N. wadsworthii*, *N. canis*, *Neisseria zoodegmatis*, *Neisseria dentiae*, *Neisseria shayeganii* and *Neisseria dumasiana*.

**Fig. 2:**
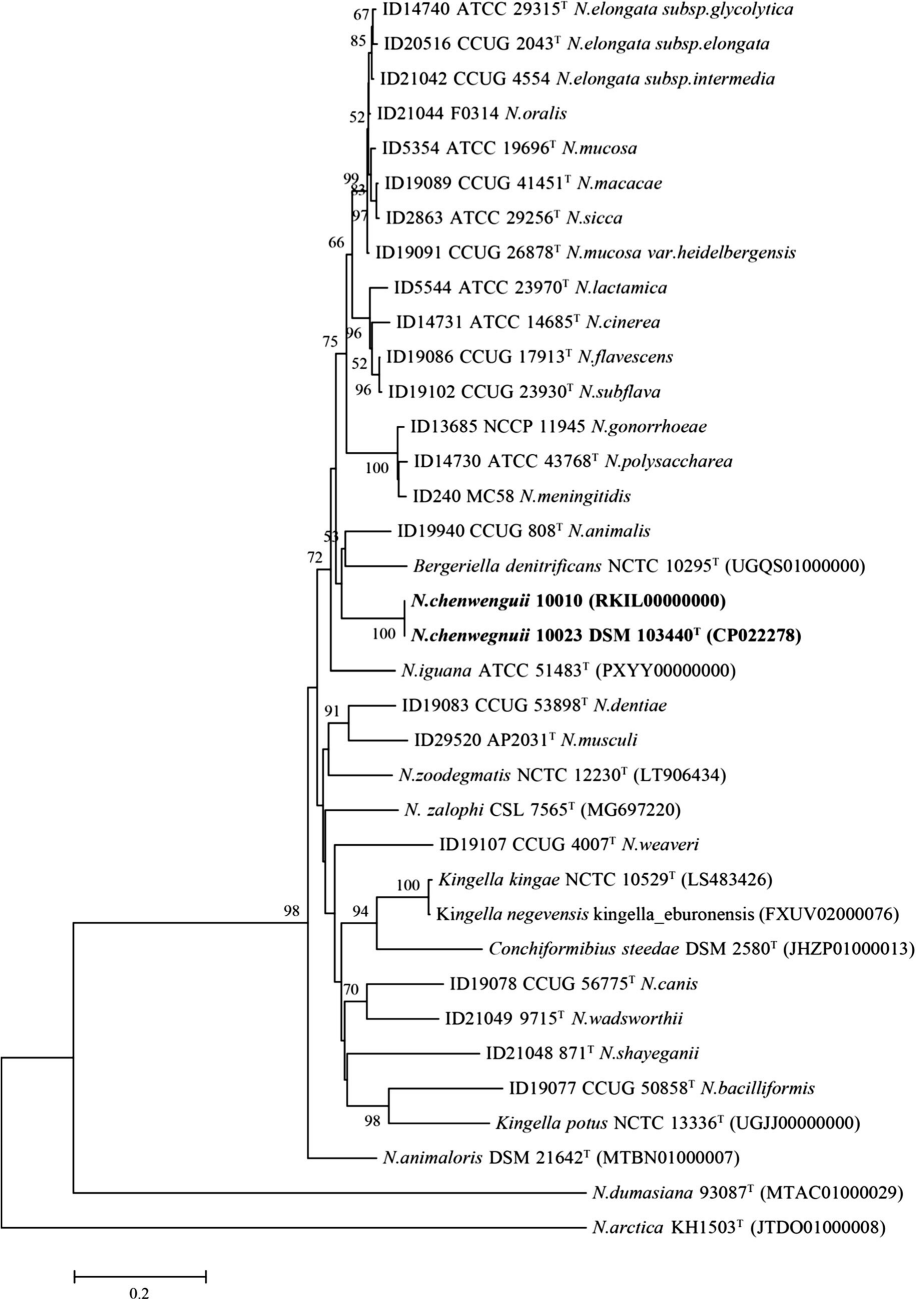
Neighbor-Joining phylogenetic tree based on *rplF* gene for the two isolates of *Neisseria chenwenguii* sp. nov. and 34 additional taxa with a total of 468 positions in the final dataset. The percentage of replicate trees in which the associated taxa clustered together in the bootstrap test (1000 replicates) are shown next to the branches. The tree is drawn to scale, with branch lengths in the same units as those of the evolutionary distances used to infer the phylogenetic tree. The evolutionary distances were computed using the Maximum Composite Likelihood method and are in the units of the number of base substitutions per site

Despite significant 16S rRNA gene sequence difference between these two novel strains and their relatives, to precisely clarify the taxonomic position, phylogenetic analysis based on the *rplF* gene was performed, which showed that these two novel isolates reside in a well-supported branch and can be easily discriminated from phylogenetically closely related species (Fig. 2).

### Average nucleotide identity and genes similarity analysis

The ANI values between strain 10023^T^ and other species in the genus *Neisseria* ranged from 73.5-79.3% (Table S1), lower than the 95% threshold, which supports the conclusion that these two isolates belong to a novel species. Moreover, the ANI value between isolates 10023^T^ and 10010 was 99.7%, further proving they belong to the same species.

The circular chromosome of 10023^T^ is 2,496,444 bp in size with an overall G+C content of 54.0 mol %. The genome of 10023^T^ encompasses 2332 protein coding sequences, 58 tRNAs and 12 rRNAs, with four copies each of the 16S, 23S and 5S rRNA genes. As predicted by the virulence factor database VFDB (http://www.mgc.ac.cn/) (Chen et al. 2016) and verified by Blast searches at NCBI, the genome of type strain 10023^T^ possesses typical *Neisseria* genes, including genes encoding putative virulence-associated factors, such as CapC, catalase, lipopolysaccharide heptosyltransferase II, lipopolysaccharide heptosyltransferase I, LptA and LptB. The similarities of these virulence-associated factors between isolate 10023^T^ and other *Neisseria* spp were in the range of 53.0–61.3%, 81.9–90.8%, 78.6–83.9%, 71.8–77.1%, 61.3–73.6% and 84.3–87.7%, respectively.

The genes similarities of *rplF*, *gyrB*, *rpoB*, *recA*, *argF*, *rpoD*, *polI*, *ribII*, *aspS* and *por* porin precursor between the type strain 10023^T^ and its closely related species (*N. zalophi* CSL 7565^T^, *N. wadsworthii* 9715^T^ and *N. canis* ATCC 14687^T^) ranged from 73.0 to 86.4% (Table 1), which clearly demonstrates that isolate 10023^T^ is different from its relatives. The similarities of the *rplF*, *gyrB*, *rpoB*, *recA*, *argF*, *rpoD*, *polI*, *ribII*, *aspS* and *por* sequences between strains 10023^T^ and 10010 are 100.0%, 99.8%, 100.0%, 99.9%, 98.5%, 99.9%, 99.9%, 100.0%, 99.9% and 100.0%, respectively.

**Table 1:** Similarities (%) for the *rplF*, *gyrB*, *rpoB*, *recA*, *argF*, *rpoD*, *polI*, *ribII*, *aspS* and *porin precursor* genes between *Neisseria chenwenguii* 10023^T^ and closely related *Neisseria*-type strains

Strain	Similarity (%) with type strain 10023^T^									
	*rplF*	*gyrB*	*rpoB*	*recA*	*argF*	*rpoD*	*polI*	*ribII*	*aspS*	*porin precursor*
*Neisseria zalophi* CSL 7565^T^	80.0	77.9	79.1	81.5	73.6	78.7	78.5	74.6	84.2	70.6
*Neisseria wadsworthii* 9715^T^	77.6	75.7	79.2	80.1	78.7	76.6	82.7	73.0	85.8	71.7
*Neisseria canis* ATCC 14687^T^	75.0	78.2	80.7	82.5	78.1	81.0	83.0	73.1	86.4	70.0

The DNA uptake sequence (DUS), a highly repetitive genomic element, plays important roles in the process of DNA transformation that ensures that all *Neisseria* species are competent. A 12-base sequence termed agDUS is substantial in the genomes of *Neisseria subflava*, *Neisseria flavescens*, *Neisseria mucosa*, *Neisseria bacilliformis* and *Neisseria weaver* (Frye et al. 2013; Mell and Redfield 2014). There are over 4000 copies of the agDUS in the 10023^T^ genome as analysed by WebLogo and EMBOSS (Crooks et al. 2004; Rice et al. 2000). This also supports the conclusion that the novel species is a member of the genus *Neisseria*.

### Chemotaxonomic characterisation

C_16:0_ and summed feature 3 (C_16:1ω7c_/C_16:1ω6c_) were identified as the predominant fatty acids in strains 10023^T^ and 10010, representing about 31.6%/29.5% and 34.9%/29.7% of the total fatty acids, respectively (Table 2). The major cellular fatty acids present in *N. zalophi*, *N. wadsworthii* and *N. canis* were C_16:0_/C_16:1ω9c_, C_16:0_/C_18:1ω7c_/summed feature 3 (C_16:1ω7c_/C_16:1ω6c_) and C_16:0_/C_18:1ω7c_, respectively. Hence, these two novel isolates can be differentiated from their close relatives by their unique fatty acids profiles (Table 2).

**Table 2:** The cellular fatty acid profiles (%) of *Neisseria chenwenguii* sp. nov. and its relatives

Fatty acid	1	2	3	4	5
C_12:0_	6.6	9.5	0.6	6.2	8.9
C_14:0_	4.1	3.7	0.7	2.5	2.2
C_16:0_	**31.6**	**29.5**	**39.2**	**29.4**	**36.2**
C_18:0_	1.1	1.9	1.0	1.6	3.1
C_16:1_ω9c	ND	ND	**38.8**	ND	ND
C_18:1_ω7c	6.8	7.2	0.3	**26.1**	**23.9**
C_18:1_ω9c	2.2	1.9	**14.9**	1.5	2.0
Summed feature 2^a^	9.6	12.1	ND	2.7	4.7
Summed feature 3^a^	**34.9**	**29.7**	ND	**23.1**	7.6

Strains: 1, 10023^T^; 2, 10010; 3, *Neisseria zalophi* CSL 7565^T^ (Volokhov et al. 2018); 4, *Neisseria wadsworthii* WC05-9715^T^; 5, *Neisseria canis* ATCC 14687^T^. Values are percentages of total fatty acids. The two or three most abundant fatty acids for each isolate are in bold

*ND* not detected

^a^Summed features consist of groups of two or three fatty acids that cannot be separated using the MIDI System. Summed feature 2 is composed of C_14:0 3_-OH/isol-C_16:1_; summed feature 3 is composed of C_16:1_ω7c/C_16:1_ω6c

### Morphological, physiological and biochemical analysis

Cells were observed to be Gram-negative, non-motile, piliated coccoid without flagella (Supplementary Fig. S2), in single, pairs, or chains, 0.2-0.9 um in diameter under transmission electron microscope. Growth occurs at 22–40 °C (optimum at 37 °C) on BHI agar with 5% defibrinated sheep blood. Growth was also observed on nutrient agar, BHI, Columbia blood agar or chocolate plates, but not on MacConkey agar after 7 days. Further, cells were found to grow in the presence of 0–4% (w/v) NaCl (optimum in 1% (w/v) NaCl) and at a pH range of 6.0-8.0 (optimum at pH 7.0). These two novel isolates can grow under anaerobic conditions and are negative for utilisation of Simmon’s citrate. The cells produce catalase and cannot reduce nitrate to nitrite. Strain 10010 was found to be positive for esterase (C4), esterase lipase (C8), naphthol-AS-BI-phosphohydrolase, proline arylamidase and α-galactosidase, which discriminates this strain from the type strain 10023^T^ (Table 3).

**Table 3:** Characteristics that differentiate *Neisseria chenwenguii* sp. nov. from closely related *Neisseria* species

Characteristic	1	2	3	4	5
Urea	+	+	−	−	−
Nitrate	−	−	+	+	+
pH range	6–8	6–8	ND	6–10	6–10
Growth in presence of NaCl (%)	4	4	6	2	2
Anaerobic growth	+	+	−	+	+
API ZYM					
Alkaline phosphatase	+	+	−	−	−
Esterase (C4)	−	+	+	+	+
Esterase lipase (C8)	−	+	+	−	+
Valine arylamidase	−	−	+	−	−
Cystine arylamidase	−	−	+	−	−
Acid phosphatase	+	+	+	−	−
Naphthol-AS-BIphosphohydrolase	−	+	+	+	+
α-Galactosidase	−	+	−	−	−
β-Galactosidase	+	+	−	−	−
API NH					
Glucose	+	+	−	+	+
Fructose	+	+	−	+	−
Maltose	+	+	−	+	−
Sucrose	+	+	−	+	+
Urease	+	+	−	−	−
Proline arylamidase	−	+	+	+	+
γ-Glutamyltransferase	+	+	−	+	−

Strains: 1, 10023^T^; 2, 10010; 3, *Neisseria zalophi* CSL 7565^T^ (Volokhov et al. 2018); 4, *Neisseria wadsworthii* WC05-9715^T^; 5, *Neisseria canis* ATCC 14687^T^

+ positive, − negative

Some key biochemical features of the novel species are helpful in distinguishing it from closely related taxa, which can be summarised as follows: (i) the novel species is positive for urea utilisation and cannot reduce nitrate to nitrite, which differentiates it from *N. zalophi*, *N. wadsworthii* and *N. canis*; (ii) according to the reaction patterns using API NH, the novel species can ferment fructose and maltose, and is urease-positive; (iii) the novel species can be distinguished from closely related species based on the reaction of alkaline phosphatase and β-galactosidase (positive for these two novel isolates) using API ZYM.

However, when characterising potential new species to the genus *Neisseria*, membership of closely related genera should be excluded. The presence of catalase activity and the absence of motility observed in these two novel isolates exclude membership in the genera *Bergeriella*, *Eikenella* and *Kingella*, which are catalase-negative. Our phylogenetic analysis based on the genes for 16S rRNA and *rplF*, and ANI analysis, showed that the novel species can be easily discriminated from all phylogenetically close relatives and other species in genus *Neisseria*.

Taken together, results from biochemical tests, fatty acid analyses, ANI and phylogenetic analyses based on the 16S rRNA and *rplF* genes all support the conclusion that isolates 10023^T^ and 10010 belong to the genus *Neisseria* and deserve classification as a novel species within the genus, for which the name *Neisseria chenwenguii* sp. nov. is proposed, with strain 10023^T^ as the type strain.

### Description of *Neisseria chenwenguii* sp. nov.

*Neisseria chenwenguii* (chen.wen.gui’i. N.L. gen. n. *chenwenguii* in honour of Professor Wengui Chen, an academician who studied acute infectious disease prevention and control in the 1950s in China).

Strains are moist, small, greyish, convex with regular margins, 0.6–1.0 mm in diameter after 48 h of growth at 37 °C under 5% CO_2_ on BHI agar with 5% defibrinated sheep blood. Optimal temperature is 37 °C. Negative for Simmons’ citrate utilisation and indole production, and positive for urea utilisation. In the API ZYM system, positive for alkaline phosphatase, leucine arylamidase, acid phosphatase and β-galactosidase. In the API NH gallery can produce acid from glucose, fructose, maltose and sucrose, and is positive for urease and γ-glutamyltransferase. The main cellular fatty acids are C_16:0_ and C_16:1ω7c_/C_16:1ω6c_. The G + C content of the type strain is 54.0 mol %.

The type strain, 10023^T^ (= DSM 103440^T^ = CGMCC 1.15736^T^), was isolated from the rectal content of a wild plateau pika (*Ochotona curzoniae*) on the Qinghai–Tibet Plateau, China. The GenBank accession numbers for the 16S rRNA gene sequences of strains 10023^T^ and 10010 are KX088452 and KX101083, respectively. The GenBank/EMBL/DDBJ accession numbers for the genome sequences of isolates 10023^T^ and 10010 are CP022278 and RKIL00000000, respectively.

## Electronic supplementary material

Below is the link to the electronic supplementary material.

Supplementary material 1 (DOCX 685 kb)

## Abbreviation

ANI: Average nucleotide identity
