# Supplementary material for: Neisseria chenwenguii sp. nov. isolated from the rectal contents of a plateau pika (Ochotona curzoniae)
Source: Antonie Van Leeuwenhoek. 2019 Feb 24;112(7):1001–10. doi: 10.1007/s10482-019-01234-2 (PMC6546665; doi:10.1007/s10482-019-01234-2)
Supplement: Supplementary file 1 — Supplementary material 1 (DOCX 685 kb) [file 10482_2019_1234_MOESM1_ESM.docx]

**Supplementary Materials**

***Neisseria chenwenguii* sp. nov. isolated from the intestinal contents of plateau pika (*Ochotona cruzoniae*)**

*Antonie van Leeuwenhoek*

Gui Zhang · Jing Yang · Xin-He Lai · Shan Lu · Dong Jin · Ji Pu · Xiangning Bai · Xuelian Luo · Yanwen Xiong · Ying Huang · Cuixia Chen · Jianguo Xu

Corresponding author: [xujianguo@icdc.cn](mailto:xujianguo@icdc.cn)

State Key Laboratory of Infectious Disease Prevention and Control, Collaborative Innovation Center for Diagnosis and Treatment of Infectious Diseases, National Institute for Communicable Disease Control and Prevention, Chinese Center for Disease Control and Prevention, Beijing 102206, People's Republic of China

Shanghai Institute for Emerging and Re-emerging Infectious Diseases, Shanghai Public Health Clinical Center, Shanghai 201508, People's Republic of China


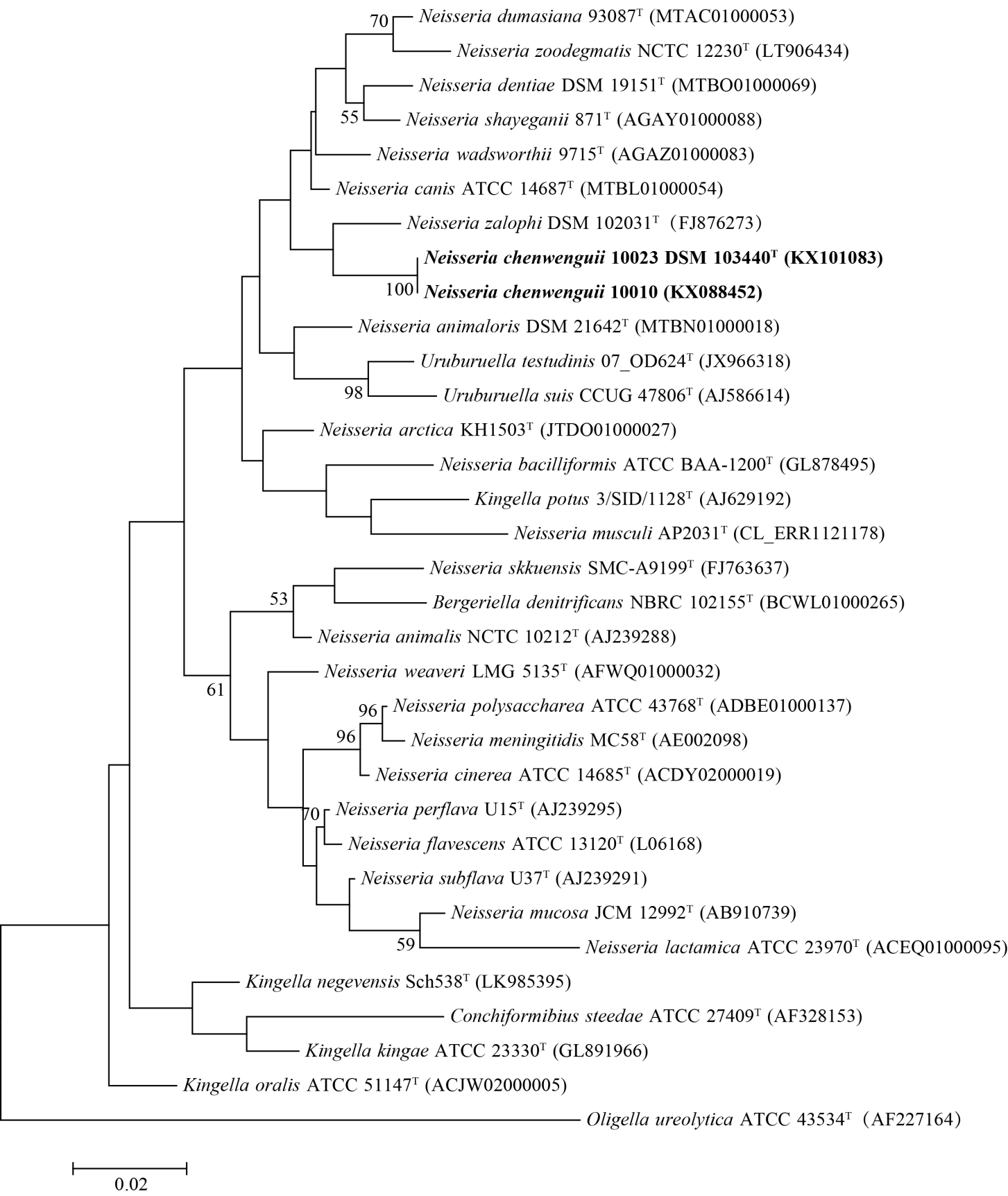


**Figure S1** Maximum Likelihood phylogenetic tree from 16S rRNA gene sequences alignment, showing the positions of strains of *Neisseria chenwenguii* sp. nov. rooted with *Oligella ureolytica* DSM 18253^T^. The percentage of trees in which the associated taxa clustered together is shown next to the branches. Initial tree(s) for the heuristic search were obtained automatically by applying Neighbor-Join and BioNJ algorithms to a matrix of pairwise distances estimated using the Maximum Composite Likelihood (MCL) approach, and then selecting the topology with superior log likelihood value. The tree is drawn to scale, with branch lengths measured in the number of substitutions per site. Bar, 0.01 expected changes per site.

.


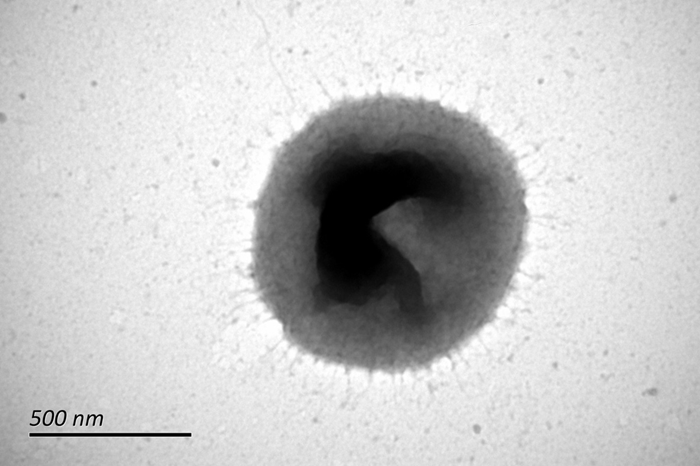


**Fig. S2** Transmission electron micrograph of strain 10023^T^ (bottom) Bar, 500 nm

**Table S1** ANI analysis between 10023^T^ and other species in the genus *Neisseria*

| Query | Reference genome | Strains | OrthoANIu  value (%) |
| --- | --- | --- | --- |
| 10023^T^ | ASM155639 | *N. perflava* CCH10-H12 | 79.3 |
| 10023^T^ | ASM22086 | *N. macacae* ATCC 33926 | 79.2 |
| 10023^T^ | RKRJ00000000 | *N. animalis* LMG 26609^T^ | 79.0 |
| 10023^T^ | ASM17465 | *N. sicca* ATCC 29256 | 78.9 |
| 10023^T^ | ASM880 | *N. meningitidis* MC58 | 78.4 |
| 10023^T^ | ASM684 | *N. gonorrhoeae* FA 1090 | 78.4 |
| 10023^T^ | ASM19629 | *N. lactamica* 020-06 | 78.4 |
| 10023^T^ | ASM210859 | *N. dentiae* DSM 19151 | 78.3 |
| 10023^T^ | ASM17673 | *N. polysaccharea* ATCC 43768 | 77.8 |
| 10023^T^ | 186165 | *N. mucosa* C102 | 77.8 |
| 10023^T^ | ASM17395 | *N. subflava* NJ9703 | 77.8 |
| 10023^T^ | ASM106396 | *N. bacilliformis* 203 NMEN | 77.6 |
| 10023^T^ | ASM17527 | *N. flavescens* SK114 | 77.5 |
| 10023^T^ | SM17389 | *N. cinerea* ATCC 14685 | 77.5 |
| 10023^T^ | ASM81803 | *N. elongata* subsp*.glycolytica* ATCC29315 | 77.2 |
| 10023^T^ | ASM210857 | *N. zoodegmatis* DSM 21643 | 77.1 |
| 10023^T^ | 2675903696 | *N. elongata* subsp*.elongata* ATCC 25295 | 77.1 |
| 10023^T^ | PXYY00000000 | *N. iguana* ATCC 51483 | 77.1 |
| 10023^T^ | ASM210854 | *N. dumasiana* | 76.9 |
| 10023^T^ | ASM210860 | *N. animaloris* DSM 21642 | 76.7 |
| 10023^T^ | 34903 D01 | *N. weaveri* 34903 D01 | 75.9 |
| 10023^T^ | ASM22687 | *N. shayeganii* 871 | 75.3 |
| 10023^T^ | MTBL00000000 | *N. canis* ATCC 14687^T^ | 75.2 |
| 10023^T^ | AGAZ00000000 | *N. wadsworthii* WC05-9715^T^ | 74.8 |
| 10023^T^ | ASM102786 | *N. arctica* KH1503 | 73.5 |
